# Study on the association between pregnancy-associated plasma protein-A and acute cerebral infarction

**DOI:** 10.3389/fneur.2023.1255714

**Published:** 2023-11-23

**Authors:** Sihang Zheng, Mohammad Showkat Hossain, Hongliang Wu, Jianfei Nao

**Affiliations:** ^1^Department of Neurology of the Second People’s Hospital of Nantong, Nantong, Jiangsu, China; ^2^Second Clinical College, China Medical University, Shenyang, Liaoning, China; ^3^Department of Orthopedics, Fuzhou Second Hospital, Fuzhou, Fujian, China; ^4^Department of Neurology, Shengjing Hospital of China Medical University, Shenyang, Liaoning, China

**Keywords:** PAPP-A, acute cerebral infarction, atherosclerosis, TOAST classification, NIHSS score

## Abstract

**Objective:**

We aimed to study the correlation between pregnancy-associated plasma protein-A (PAPP-A) and acute cerebral infarction (ACI).

**Methods:**

Patients who had the symptoms of paralysis, aphasia, or sudden neurological impairment from June 2020 to October 2021 were chosen. There were 159 patients diagnosed with ACI as the experimental group and 102 patients without ACI as the control group. We collected clinical data and observed whether they have a certain impact on plasma PAPP-A levels. The ACI group was divided into two groups: mild neurological deficit group (NIHSS score < 3) and moderate and severe neurological deficit group (NIHSS score > 3). The ACI group was divided into the atherosclerotic-type group and the arteriolar occlusion-type group according to the TOAST classification. The ACI group was divided into a good prognosis group (mRS ≤ 2 points) and a poor prognosis group (mRS > 2 points) using the Modified Rankin Scale (mRS) for 90 days of follow-up. Plasma PAPP-A levels were compared between those groups.

**Results:**

(1) The plasma PAPP-A level in patients with ACI (1.840 ± 0.281) was significantly higher than that in the control group (1.690 ± 0.260). Smoking history, leukocyte count, cystatin C, homocysteine, and plasma PAPP-A levels were independently correlated with ACI. (2) The level of PAPP-A in patients with moderate and severe neurological impairment was lower than that in patients with mild neurological impairment. (3) The level of PAPP-A in patients in the arteriolar occlusion-type group was higher than that in patients in the atherosclerosis-type group. (4) The PAPP-A levels in the group with elevated low-density lipoprotein are higher than those in the group with normal low-density lipoprotein. (5) Plasma PAPP-A level was not correlated with infarction location, infarction volume, or prognosis at the 90-day follow-up.

**Conclusion:**

(1) The level of plasma PAPP-A could be the independent risk factor of ACI. It is positively correlated with triglyceride and cholesterol content. (2) PAPP-A level is positively correlated with low-density lipoprotein. (3) PAPP-A levels between different disease severities have a significant difference. (4) The level of plasma PAPP-A in the arteriolar occlusion-type group was higher than that in the atherosclerotic-type group.

## 1. Introduction

Acute cerebral infarction (Acute Cerebral Infarction, ACI) refers to a group of diseases caused by thrombosis of intracranial blood vessels or occlusion of blood vessels (1, 2). Because of the non-renewable nature of nerves, it seriously endangers human health. This disease mainly threatens the life and quality of life of people over 60 years of age, and it is a disease with a high fatality rate. The most common symptoms are speech disorder and unilateral limb movement disorder (3). Patients with acute cerebral infarction recover slowly after the disease. Some of them will be left with sequelae such as limb movement disorder, drinking water cough, dysphagia, and aphasia to varying degrees, among which 5–15% of patients will even have secondary epilepsy (4). As a result, the quality of life and self-care ability of some patients with sequelae or secondary epilepsy will decrease significantly (5). Stroke has now become the most common cause of death in China (6). Fast, convenient, and accurate prediction of the occurrence of acute cerebral infarction helps prevent further brain tissue damage, improves the disease’s prognosis, and significantly reduces the mortality and disability rate of acute cerebral infarction. Therefore, this study aimed to explore accurate and specific biomarkers for the occurrence and development of cerebrovascular diseases.

Studies have shown that the leading causes of acute cerebrovascular disease include atherosclerosis, hemodynamic changes, abnormal glucose and lipid metabolism, and cardiovascular diseases (7). The most common factors are atherosclerosis and hemodynamic abnormalities. In the process of disease diagnosis, evaluation of curative effect, and prognosis, biochemical blood markers are convenient to operate and low in price, so they are widely used. PAPP-A has recently been found to cause plaque instability and began to be included in cerebrovascular disease-related studies (8).

PAPP-A is a glycoprotein with 1,547 amino acid residues that was first extracted from a pregnant woman’s serum in 1974 by Lin et al. (9). Later, Oxvig, Schulz O, and others (10, 11) found that it has two forms in the human body.

One is that two units of PAPP-A bind two units of Proform of eosinophil major basic protein (ProMBP) to form a heterotetramer (PAPP-A/proMBP). This structure is the main form of PAPP-A present in the human body. The second is that it exists within unstable patches as a homodimer, rarely enters the circulatory system, and is less abundant. Because each molecule contains a zinc ion binding site on PAPP-A, this site is similar to the functional structure of Matrix Metalloproteinases (MMP). Therefore, some scholars believe that PAPP-A may belong to a member of the large family of matrix metalloproteinases and participate in regulating glucose and lipid metabolism in the human body (12). Its mechanism of action is the degradation and remodeling of the extracellular matrix (ECM), which can degrade vascular components and weaken the fibrous cap connective tissue, leading to the transformation of the plaque into an unstable state. After further plaque rupture, the embolus falls off and plugs into blood vessels, leading to stroke (8).

Previous PAPP-A-related studies mainly focused on pregnancy-related diseases such as placental abruption (13), gestational diabetes (14), eclampsia (15), and acute coronary syndrome (16) as well as various organ ischemia and hypoxic diseases caused by vascular endothelial injuries. Some laboratory studies have proved that the level of PAPP-A in patients with coronary heart disease is significantly higher compared to ordinary people, and it is obviously positively correlated with the degree and severity of lesions (16), which can be used as a predictive index of coronary heart disease. There are few relevant explorations between PAPP-A and ischemic stroke. This study intends to explore the relationship between PAPP-A and acute ischemic stroke by studying the different aspects of the incidence, risk factors, severity, classification, and short-term prognosis of acute ischemic stroke.

## 2. Data and methods

### 2.1. Study subjects

#### 2.1.1. Case group

A total of 159 patients were diagnosed with acute cerebral infarction in the neurology Department of Shengjing Hospital of China Medical University from June 2020 to October 2021. All the patients included met the diagnostic criteria for acute ischemic stroke (17), which include must have happened within 72 h, with speech disorders, central facial tongue paralysis, symptoms and clear physical signs of nerve function defects such as numbness or disability of one limb, and confirmed by head CT or MRI with new cerebral infarction. The patients were classified as large atherosclerosis or small artery infarction type according to TOAST classification (18). The exclusion criteria were diseases over 72 h, venous sinus thrombosis, cerebral hemorrhage and other vascular diseases, cardiac insufficiency, atrial fibrillation, acute myocardial infarction, severe liver and kidney function reduction, trauma, infection, and other testing indicators. A total of 86 men and 73 women were included, with a mean age of (64.61 ± 12.76).

#### 2.1.2. Control group

Health examination patients and patients hospitalized during the same period diagnosed with benign positional vertigo, facial neuritis, and vestibular neuronitis were selected.

Patients with ACI were excluded by MRI + DWI. A total of 102 patients were obtained. Poisoning, malignancy, hematological disorders, and autoimmune diseases were excluded, as well as patients with drug abuse. There were 50 men and 52 women. The mean age was (65.39 ± 10.95) years.

### 2.2. Methods

#### 2.2.1. Collection of the clinical data

Clinical data of the enrolled patients after their admission were collected. General data included age, gender, past history (having stroke-related risk factors such as hypertension, diabetes, cardiovascular disease, and atherosclerosis), personal history (smoking history, drinking history, drug abuse, etc.), contact information, and admission time. Laboratory data included white blood cell and platelet count, liver function, kidney function, blood glucose and lipid series, glycated hemoglobin A1c, five items of coagulation, liver function, lactate dehydrogenase, blood homocysteine and anemia series, and other indicators. Imaging data included head CT, MRI, and carotid artery color ultrasound.

#### 2.2.2. Group of clinical data

Patient disease severity at admission was assessed using the National Institute of Health Stroke Scale (NIHSS) score and infarction volume. After admission, they were divided into two groups according to their medical history and physical examination after completing the NIHSS score (19). Those with an NIHSS score of three were those with mild neurological deficits, and those with an NIHSS score > 3 had moderate to severe neurological deficits. According to the admission imaging findings (head CT or MRI), the anterior and posterior circulation infarctions were differentiated according to whether the blood vessels of the infarction site and the corresponding imaging examination corresponded to them. The infarction volume was calculated using the Pullicino formula (20) to determine whether the infarction volume was less than 5 cm^3^. Patients with acute ischemic stroke were classified into the large area cerebral infarction (>5 cm^3^) group and the small size cerebral infarction group (5 cm^3^). The group of cerebral infarction was performed using TOAST classification and divided into a large artery atherosclerosis group and a small artery occlusion group (after evaluating the clinical data of patients in this study, only patients with extensive artery atherosclerosis and small artery occlusion cerebral infarction were selected). The prognosis of patients was assessed using the modified Rankin scale (mRS) by telephone follow-up 90 days after the onset. They were divided into a good prognosis group (mRS ≤ 2) and a poor prognosis group (mRS > 2) based on whether the mRS score was greater than two (21). According to the results, they were further subdivided into different subgroups through glucose and lipid metabolism and coagulation indexes.

#### 2.2.3. Specimen collection and testing

Using the EDTA anticoagulant tube, 3–5 mL of fasting elbow venous blood was taken in the morning of the next day after admission (the blood collection time of the experimental group should be less than 72 h). The upper plasma was isolated by centrifugation at 3,000 r for 15 min within 48 h, transferred to the EP tube, and then cryopreserved in a refrigerator at 80°C. After collecting specimens, plasma PAPP-A levels were detected using the ELISA kit of human PAPP-A produced by Shanghai Enzyme-linked Biotechnology Co., Ltd.

#### 2.2.4. Statistical analysis

Experimental data were processed statistically using SPSS25 statistical software. The mean ± standard deviation expressed the measurement data conforming to the normal distribution. The interquartile spacing is applied if the data does not conform to the normal distribution. For continuous and normal distribution data, two independent sample *T*-tests were performed. For data not conforming to the normal distribution, non-parametric tests (Mann–Whitney *U-*test) were used. Count data were expressed as a percentage. Differences between the two groups were calculated using the chi-square test. Binary logistic regression analysis was performed for indicators with the statistical difference between the ACI and control groups. According to different data types, Pearson or Spearman tests were used for linear correlation analysis. When analyzing the results, if the probability was *p* < 0.05, it was considered that the difference was related. All inspection methods were two-sided.

## 3. Results

### 3.1. Analysis of general data results

The average age of the case group was (64.61 ± 12.76) years, including 86 men and 73 women. The average age of the control group was (65.39 ± 10.95) years, including 50 men and 52 women. The comparison of age and gender factors between the two groups indicated that *p* > 0.05, which means the two groups were comparable. After general data comparison, the proportion of smoking history and drinking history in the case group was significantly higher than in the control group. The white blood cell count, cystatin C, blood homocysteine value, coagulation-related indicators, lactate dehydrogenase, and plasma PAPP-A levels in the case group were higher than in the control group. The difference was statistically significant (*p* < 0.05; Table 1).

**Table 1:** General data comparison between the case group and control group.

Index	Case group (*n* = 159)	Control group (*n* = 102)	T/Z/x2 values	*P*
Age (year)	64.63 ± 12.76	65.39 ± 10.95	0.496	0.620
Male [*n* (%)]	86(54.08%)	50(49.01%)	1.160	0.281
Smoking history [*n* (%)]	72(45.28%)	31(20.19%)	6.042	0.014
Drinking history [*n* (%)]	51(32.08%)	21(20.59%)	4.284	0.038
Hypertension [*n* (%)]	94(59.12%)	52(50.98%)	1.889	0.169
Diabetes mellitus [*n* (%)]	40(25.16%)	33(32.35%)	1.473	0.225
Atrial fibrillation [*n* (%)]	14(8.81%)	4(3.92%)	2.336	0.124
Coronary heart disease [*n* (%)]	18(11.32%)	16(15.69%)	0.983	0.322
Leucocyte count (*109/L)	6.63(2.68)	6.28 ± 1.85	−2.335	0.020
Platelet count (*109/L)	212.5(78)	208.03 ± 59.63	−0.648	0.517
HbA1c (%)	6.10(1.5)	6.05(1.4)	−0.272	0.786
Triglyceride (mmol/L)	1.30(0.95)	1.44(1.00)	−0.614	0.539
Total cholesterol (mmol/L)	4.42 ± 1.01	4.49 ± 1.11	0.474	0.636
LDL (mmol/L)	2.73(1.44)	2.73 ± 0.96	−0.691	0.489
HDL (mmol/L)	1.02(0.4)	1.07(0.41)	−1.300	0.193
Urea (μmol/L)	5.10(2.31)	5.28(1.88)	−0.826	0.409
Uric acid (μmol/L)	304.71 ± 103.02	297.95 ± 85.23	−0.447	0.665
Creatinine (μmol/L)	62.15(21.22)	63.07 ± 16.38	−0.634	0.526
Cystatin C (mg/L)	1.17(0.32)	1.04(0.26)	−3.012	0.000
Homocysteine (μmol/L)	12.05(1.12)	10.33(4.37)	−3.574	0.000
Vitamin B12 (pg/mL)	250.2(125)	261(205)	−0.583	0.560
Plasma prothrombin time	11.3(1.45)	10.9(0.9)	−4.238	0.000
Partial thromboplastin time	32(5.5)	30(3.5)	−2.592	0.010
Thrombin time	16.1(0.9)	16.7(0.9)	−6.766	0.000
Fibrinogen	2.9(0.95)	2.7(0.9)	−3.245	0.000
D-dimer	134(175)	90(112.5)	−3.043	0.000
Glutamic-pyruvic transaminase	18(15.5)	16(12)	−0.253	0.800
Lactate dehydrogenase	185(66)	181(49)	−2.056	0.040
PAPP-A (ug/mL)	1.874 ± 0.270	1.69 ± 0.26	5.291	0.000

Since plasma PAPP-A was originally secreted from women’s reproductive organs, the case and control groups were grouped according to gender. There was no significant difference in plasma PAPP-A concentration between the case and control groups (*p* = 0.246 *p* = 0.116). Excluding the influence of gender factors, the PAPP-A concentration levels in both groups were still statistically significant (the *p*-value was less than 0.05; Table 2). Therefore, it can be proved that the PAPP-A level is not affected by gender factors in this experiment.

**Table 2:** Comparison of the case and control groups according to gender.

PAPP-A (ug/mL)	Male	Female	T/Z/x2 values	*P*
Case group	0.8641 ± 0.275	0.8112 ± 0.286	1.165	0.246
Control group	1.735 ± 0.331	1.651 ± 0.164	1.588	0.116
T/Z/x2 values	−3.300	−4.214		
*P*	0.001	0.000		

Plasma PAPP-A was initially found in pregnant women. Many studies have proved that plasma PAPP-A correlates with glucose and lipid metabolism and coagulation indexes in women. Therefore, Pearson correlation analysis was used to analyze the relationship between glucose and lipid metabolism, coagulation-related indexes, and plasma PAPP-A levels in the case group and control group members recorded in this experiment. The analysis results suggested a significant linear positive relationship between human triglycerides, cholesterol content, and plasma PAPP-A levels (Pearson correlation > 0, *p* < 0.05; Table 3).

**Table 3:** Correlation analysis of glucose and lipid metabolism, coagulation indexes, and plasma PAPP-A level.

Factor	Pearson correlation	*P*
PT	0.012	0.690
APTT	−0.010	0.869
FIB	0.088	0.166
TT	0.010	0.879
DD	0.018	0.775
Diabetes mellitus	−0.090	0.150
Glycosylated hemoglobin	0.014	0.855
Glycerin trilaurate	0.156	0.049
Cholesterol	0.208	0.010
HDL cholesterol	−0.064	0.317
LDL cholesterol	0.042	0.513

### 3.2. Analysis of the independent influencing factors of acute cerebral infarction

Through SPSS, we used binary logistic regression to analyze the independent influencing factors of the experimental data.

In the comparison of general data between the ACI group and control group, statistically significant factors (the proportion of smoking history and drinking history, coagulation-related indexes, white blood cell count, cystatin C, blood homocysteine value, and plasma PAPP-A level) were introduced into the regression equation for stepwise regression.

The final results showed that partial thromboplastin time, fibrinogen, blood homocysteine value, and plasma PAPP-A level were independent risk factors for acute cerebral infarction (OR > 1, *p* < 0.05; Table 4).

**Table 4:** A logistic regression analysis of the influencing factors of acute cerebral infarction.

Correlative factor	OR	95%CI	*P*
APTT	1.074	1.008–1.144	0.027
Fibrinogen	1.861	1.211–2.859	0.005
Homocysteine	1.047	1.003–1.092	0.015
PAPP-A	1.002	1.000–1.003	0.036

### 3.3. Association between PAPP-A level and NIHSS score

The severity of the patient’s condition is related to the prognosis and the quality of life. Therefore, the patients were grouped according to their NIHSS score within 72 h after disease onset. There were 92 cases in the group with a mild neurological deficit (NIHSS 3) and 65 cases in the group with a moderate to severe neurological deficit (NIHSS > 3). Plasma PAPP-A levels were higher in the mild neurological deficit group than in the moderate–severe neurological deficit group (*p* < 0.05; Table 5). Further analysis of the Spearman linear correlation between PAPP-A and NIHSS score did not prove the correlation (r = −0.135, *p* > 0.05).

**Table 5:** Association between PAPP-A level and the degree of neurological deficits.

	PAPP-A (ug/mL)	*P*
Group with mild neurological deficit (92 patients)	1.915 ± 0.269	0.02
Group with moderate to severe neurological deficit (65 patients)	1.815 ± 0.262	

### 3.4. Association between PAPP-A level and cerebral infarction volume

Infarction volume was calculated based on the DWI sequence of the head MRI or the CT examination at admission in the ACI group, using the Pullicino formula. They were divided into infarction volume < 5 cm^3^ (106 cases) and infarction volume ≥ 5 cm^3^ (46 cases). No statistically significant difference was found between the two groups when compared (Table 6).

**Table 6:** Association between PAPP-A level and infarction volume.

	PAPP-A (ug/mL)	*P*
Infarction volume < 5 cm^3^(106 cases)	1.826 ± 0.281	0.261
Infarction volume ≥ 5 cm^3^(46 cases)	1.882 ± 0.286	

### 3.5. Association between PAPP-A level and different TOAST classification

Stroke was classified according to the TOAST classification. According to the imaging examination results, the large atherosclerosis group (81 cases) and the small artery occlusion group (74 cases) were screened out. Comparing the PAPP-A levels of the two groups, it was found that the small artery occlusion group had a higher PAPP-A level, and the results were statistically significant (*p* = 0.002; Table 7). Further Spearman linear correlation analysis showed that they were correlated (r = −0.225, *p* = 0.005).

**Table 7:** Association between PAPP-A level and different TOAST classification.

	PAPP-A (ug/ml)	*P*
Large atherosclerosis group (81 patients)	1.777 ± 0.280	0.002
Small artery occlusion group (74 cases)	1.914 ± 0.266	

### 3.6. Association between PAPP-A level and different TOAST classification

The patients in the case group were followed up by telephone 90 days after the disease. The total number of lost cases was 14. According to the mRS score, the patients were divided into two groups: 99 patients with a good prognosis (mRS ≤ 2 points) and 46 patients with a poor prognosis (mRS > 2 points). There was no significant difference in plasma PAPP-A level between the group with good prognosis and those with poor prognosis (*p* > 0.05; Table 8).

**Table 8:** Association between PAPP-A level and prognosis.

	PAPP-A (ug/mL)	*P*
Good prognosis group (99 cases)	1.838 ± 0.284	0.931
Poor prognosis group (46 cases)	1.843 ± 0.270	

### 3.7. Association between PAPP-A level and different TOAST classification

According to the imaging results and the location of symptoms and signs, the patients with acute cerebral infarction were divided into the anterior circulation infarction group (89 cases) and the posterior circulation infarction group (66 cases). There was no significant difference in plasma PAPP-A levels between the anterior and posterior circulation infarction groups (*p* > 0.05; Table 9).

**Table 9:** Association between PAPP-A level and infarction site.

	PAPP-A (ug/mL)	*P*
Anterior circulation infarction group (89 cases)	1.875 ± 0.262	0.095
Posterior circulation infarction group (66 cases)	1.798 ± 0.301	

### 3.8. Association between PAPP-A level and different TOAST classification

Previous clinical research data have proved that the low-density lipoprotein (LDL) level in patients is closely related to the occurrence and development of acute cerebral infarction. Therefore, according to the level of serum LDL, the patients in the experimental group were divided into 71 cases in the normal LDL group and 80 cases in the elevated LDL group. Compared with the two groups, the plasma PAPP-A level in the two groups was statistically significant (*p* = 0.04; Table 10). Further Spearman correlation analysis showed a significant linear correlation between plasma PAPP-A and LDL levels (r = 0.167 *p* = 0.040).

**Table 10:** Association between PAPP-A level and LDL (1).

	PAPP-A (ug/mL)	*P*
normal LDL group (71 cases)	1.782 ± 0.316	0.040
Elevated LDL group (80 cases)	1.882 ± 0.219	

The above data showed that a high PAPP-P level tends to be more likely to diagnose small artery occlusion cerebral infarction. Therefore, according to the level of serum LDL, the patients with small artery occlusion cerebral infarction were divided into a normal LDL group (29 cases) and an elevated LDL group (32 cases). Compared with the two groups, the plasma PAPP-A levels had no significant difference between the two groups (*p* > 0.05; Table 11).

**Table 11:** Association between PAPP-A level and LDL (2).

	PAPP-A (ug/mL)	*P*
normal LDL group (29 cases)	1.901 ± 0.240	0.308
Elevated LDL group (32 cases)	1.973 ± 0.304	

The glycosylated hemoglobin level in the human body shows a patient’s blood sugar control in the past 120 days. Therefore, according to the glycosylated hemoglobin level, the patients in the case group were divided into 56 patients with well-controlled blood glucose and 56 patients with poor control. Compared with the two groups, the plasma PAPP-A level had no significant difference between the two groups (*p* > 0.05; Table 12).

**Table 12:** Association between PAPP-A level and blood glucose (1).

	PAPP-A (ug/mL)	*P*
Blood glucose well-controlled group (56 cases)	1.834 ± 0.290	0.493
Blood glucose poor-controlled group (56 cases)	1.869 ± 0.264	

The above data proved that a high PAPP-A level tends to be more likely to diagnose small artery occlusion cerebral infarction. Therefore, according to the serum glycosylated hemoglobin level of patients, the patients with small artery occlusion cerebral infarction were divided into 26 patients in the blood glucose well-controlled group and 26 patients in the blood glucose poor-controlled group. Compared with the two groups, the plasma PAPP-A level had no significant difference between the two groups (*p* > 0.05; Table 13).

**Table 13:** Association between PAPP-A level and blood glucose (2).

	PAPP-A (ug/mL)	*P*
Blood glucose well-controlled group (26 cases)	1.914 ± 0.277	0.564
Blood glucose poor-controlled group (26 cases)	1.954 ± 0.215	

### 3.9. Association between PAPP-A level and different TOAST classification

The above data proved that a high PAPP-A level tends to be more likely to diagnose small artery occlusion cerebral infarction. Hypercoagulability is a major cause of cerebrovascular disease. Therefore, the patients in the small artery occlusion cerebral infarction group were further divided into a normal group and an elevated group according to each coagulation index. There was no significant difference in plasma PAPP-A level between the two groups (P1 > 0.05, P2 > 0.05, and P3 > 0.05; Table 14).

**Table 14:** Association between PAPP-A level and coagulation index.

PAPP-A (ug/mL)/number of cases	Normal group	Elevated group	*P*
FIB	1.939 ± 0.256(55)	1.892 ± 0.267(16)	0.526
D-Dimer	1.931 ± 0.266(54)	1.920 ± 0.237(17)	0.875
Blood platelet	1.932 ± 0.263(60)	1.907 ± 0.238(8)	0.875

## 4. Discussion

Pregnancy-related plasma protein-A is a kind of metalloproteinase. Its structure contains the binding site of Zn. It was found in research that it is zinc and calcium-dependent, which was first found in the body of pregnant women (1). Nowadays, PAPP-A has been proven to be expressed in reproductive organs and embryonic appendages and secreted by bone marrow cells, fibroblasts, vascular smooth muscle cells, osteoblasts, adipose tissue, and other components (12, 22).

There are few domestic experimental and clinical studies on PAPP-A and acute cerebral infarction. At present, the leading cause of ischemic cerebrovascular disease is atherosclerosis. It leads to the stenosis of intracranial and extracranial vessels and the secondary ischemia and hypoxia injury of intracranial nerve cells. Atherosclerosis is a chronic inflammatory disease characterized by atherosclerotic plaque deposited on the artery’s wall. With the progress of the disease, it may cause the stenosis of the corresponding blood vessel or the shedding of plaque, resulting in the insufficiency of blood supply and infarction of the arterial blood supply area.

The clinical application of PAPP-A focuses on pregnancy-related diseases. With the continuous progress of research, A Bayes Genis et al. first proposed the possibility of PAPP-A as a biomarker of unstable plaque atherosclerosis in 2001. They proved the relationship between PAPP-A content and acute coronary syndrome. Later, its structure was proved to be highly similar to matrix metalloproteinases. Its main pathogenic principle was to degrade the extracellular matrix, weaken the fiber cap, and finally lead to the plaque transition from stable to unstable, eventually leading to cardiovascular and cerebrovascular events (23). With the deepening of research, the mechanism of action has been successively proved: PAPP-A mainly hydrolyzes insulin-like growth factor binding proteins (IGFBPs). Its primary physiological substrate is IGFBP-4. After hydrolysis, two fragments of NT-IGFBP-4 and CT-IGFBP-4 with lower physiological activity were formed. IGFBP-4 is a kind of IGF inhibitor, and the main principle is a high affinity for IGF-1 and IGF-II. The formed IGFBP-4/IGF-1 and IGFBP-4/IGF-2 have no physiological activity, inhibiting the interaction between IGF-I, II, and related receptors on the cell membrane. At the same time, when IGFBP-4 is combined with IGF, the rate of hydrolysis of IGFBP-4 by PAPP-A increases with a positive feedback effect (24). When the level of PAPP-A increases, the cleavage of IGFBP-4 increases, and the free IGF-I increases. Therefore, PAPP-A can enhance the utilization of IGF-I. IGF-I is a mitogen of smooth muscle cells (25), which can stimulate the migration and proliferation of smooth muscle cells, leading to endothelial proliferation and stenosis (26). PAPP-A may promote the development of atherosclerosis, plaque instability, and plaque rupture mainly through PAPP-A, IGFBP-4, and IGF-I/IGF-IR signal transduction systems, as well as interaction with other inflammatory cells and inflammatory factors (27).

This study mainly discussed whether PAPP-A has the value of predicting the incidence of acute cerebral infarction by comparing the general data and clinical data of patients with acute cerebral infarction who received medical treatment in the Department of Neurology of Shengjing Hospital of China Medical University and those who participated in a physical examination in the same period from 2020 to 2021. By comparing the PAPP-A level of different subgroups of patients with acute cerebral infarction, we evaluated whether the PAPP-A level in patients was related to the time of onset, infarct location and volume, severity of disease, and prognosis.

Comparing the general data of the ACI group and the control group showed that the plasma PAPP-A level of patients with acute cerebral infarction patients was higher than that of the control group, and the difference was statistically significant (*p* < 0.05). The factors with statistical significance in the general data were included in the binary logistic regression for further independent influencing factor analysis. The results indicated that partial thromboplastin time, fibrinogen, blood homocysteine value, and plasma PAPP-A level were independent influencing factors of acute cerebral infarction (*p*-values were less than 0.05), and they were all risk factors (OR > 1). The plasma PAPP-A level was an independent risk factor of acute ischemic stroke (*p* < 0.001). Triglycerides and cholesterol were positively correlated with PAPP-A in plasma. However, the gender of the experimental and control groups cannot prove to have an impact on the plasma PAPP-A level temporarily. It has been established that PAPP-A is mainly expressed by vascular smooth muscle cells in the middle and inner layers of cardiovascular and cerebrovascular diseases (28). In progressive plaque, PAPP-A is highly correlated with vascular endothelial cells, vascular smooth muscle cells, and macrophages. Its expression is significantly increased in late plaque, which promotes atherosclerosis (29), thus accelerating the occurrence and development of cerebral infarction.

In the course of clinical diagnosis and treatment, it is an essential step to evaluate the degree of neural function defect. Often, the degree of neurological deficit reflects the severity of the disease, which has a specific significance in indicating the quality of life, mortality, disability, and prognosis of patients with acute cerebral infarction. At present, NIHSS score (30) and infarction volume are generally recognized as evaluation methods in clinical practice. In this study, the ACI group was grouped according to NIHSS score. The results showed that the plasma PAPP-A level of patients with mild neurological impairment (NIHSS score ≤ 3) was higher than that of patients with moderate and severe neurological impairment (NIHSS score > 3), and the difference was statistically significant (*p* < 0.05). However, further Spearman correlation analysis could not prove the existence of a correlation between the two. However, previous studies have all considered that the level of PAPP-A is positively correlated with the severity of the disease. The level of PAPP-A in the moderate and severe neurological deficit group should be higher than in the mild neurological deficit group (31). It is inconsistent with the results of this experiment. It is considered that the possible cause is selectivity bias.

The selected ACI patients were divided into groups according to infarct volume. There was no significant difference in PAPP-A levels between the group with infarction volume < 2.5 cm^3^ and those with infarction volume ≥ 2.5 cm^3^. That is, the plasma PAPP-A level was not related to the infarction volume. There is no relevant evidence to prove the relationship between PAPP-A level and cerebral infarction volume, and there is a lack of comparative data. However, considering that previous studies believed that the level of PAPP-A was related to the severity of the disease, and this experiment could not prove the correlation between the two, more clinical trials were needed to prove whether it was related.

In this study, the acute cerebral infarction group patients were grouped according to the TOAST classification. The comparative results showed that the plasma PAPP-A level was higher in the arteriole occlusion group than in the large atherosclerosis group, and the results were statistically significant. This means that the level of plasma PAPP-A is associated with the stroke subtype of acute cerebral infarction.

In addition, the study divided patients into the anterior circulatory infarction group and the posterior circulatory infarction group. PAPP-A levels were compared between the two groups, and no significant difference was found. That is, it cannot be proved that different infarct sites can lead to changes in PAPP-A levels.

In this experiment, the prognosis of patients in the ACI group was also evaluated by mRS. There was no significant difference in the PAPP-A level between the poor and good prognosis. Relevant studies have confirmed that PAPP-A levels correlated with patients’ long-term prognosis at the 2-year follow-up (32). The PAPP-A levels in the poor-outcome group were significantly higher than those in the good-outcome group. The follow-up time of our experiment is 90 days. It is considered that the plasma PAPP-A level is related to long-term prognosis, but it is less related to short-term prognosis. Some scholars have proved in clinical research that IGF plays an essential role in the normal growth, development, and repair of the brain. Through RasMAPKs (Erk1,2 and p38) and PI-3 K/Akt pathway, it participates in the neurogenesis of the embryonic brain, mature neurons and adult brain, axon regeneration, and formation of new synapses. Therefore, it is speculated that PAPP-A can play a role in brain development and repair by regulating the IGF axis (33, 34). It plays a specific role in the prognosis of acute cerebral infarction.

In addition, according to the current clinical research, PAPP-A impacts the metabolism of glucose and lipids in the human body. This experiment proved that a high level of PAPP-A tended to be diagnosed as arteriolar occlusive cerebral infarction. Therefore, the patients in the experimental group’s arteriolar occlusive group were divided according to the standard value of low-density lipoprotein and glycosylated hemoglobin levels. The results showed no significant difference between plasma PAPP-A levels and glucose and lipid metabolism levels in patients with arteriolar occlusion. Therefore, it can be considered that the glucose and lipid metabolism level does not affect the plasma PAPP-A level in patients with arteriolar occlusion.

Finally, considering that plasma PAPP-A was initially found in pregnant women, one of the significant characteristics of pregnant women was blood hypercoagulability, and one of the major causes of arteriolar occlusive cerebral infarction was hemodynamic changes. Therefore, according to coagulation indexes, the patients with TOAST classification of arteriolar occlusion in this experimental group were divided into a standard group and an abnormal group. The analysis showed no significant difference in plasma PAPP-A levels between the two groups, which was inconsistent with the expected results. It may be that the blood coagulation mechanism of patients with arteriolar occlusion needs to be more precise, or the selective bias of patients admitted this time led to inaccurate data results.

To sum up, this study confirmed that the plasma PAPP-A level of ACI patients at admission was higher than that of the control group. The level of PAPP-A is one of the independent risk factors for the onset of cerebral infarction, which has a specific value for predicting the onset of cerebral infarction. The plasma PAPP-A level in the mild neurological deficit group was higher than that in the moderate and severe neurological deficit groups, which was statistically significant. PAPP-A was not related to the size and location of the infarction but to the TOAST classification. The plasma PAPP-A level in patients with small artery infarction is higher than that in the atherosclerotic group, and there is a correlation. Patients with high levels of PAPP-A in the body are more likely to have cerebral infarction of arteriolar infarction type. The plasma PAPP-A level in the group with a poor prognosis was not significantly different from that in the group with a good prognosis. Therefore, plasma PAPP-A level is expected to become one of the indicators indicating the onset, attack type, and severity of ACI patients.

The limitation of this study is that the number of patients in the experimental and the control groups is limited. They all come from the inpatients in the Department of Neurology in our hospital from 2020 to 2021. There is a certain degree of selective bias.

## 5. Conclusion

1. Plasma levels of PAPP-A were higher in the ACI group. It was an independent risk factor for the onset of acute cerebral infarction. Furthermore, it positively correlated with the triglyceride and cholesterol content in patients.
2. Plasma PAPP-A levels were associated with the disease severity of acute cerebral infarction. PAPP-A levels were higher in less severe diseases.
3. Plasma PAPP-A levels were associated with the TOAST typing of cerebral infarction. High levels of PAPP-A suggested a high probability of arteriolar infarct type. Moreover, glucose and lipid metabolism levels in patients with arteriolar infarction did not affect PAPP-A levels.
4. Plasma PAPP-A levels were not associated with cerebral infarction’s size, location, and short-term prognosis.

## Data availability statement

The raw data supporting the conclusions of this article will be made available by the authors, without undue reservation.

## Ethics statement

The studies involving humans were approved by the Ethics Committee of Shengjing Hospital of China Medical University (2022PS715K). The studies were conducted in accordance with the local legislation and institutional requirements. The participants provided their written informed consent to participate in this study.

## Author contributions

SZ: Conceptualization, Data curation, Formal analysis, Funding acquisition, Investigation, Methodology, Project administration, Resources, Software, Supervision, Validation, Visualization, Writing – original draft, Writing – review & editing. MH: Conceptualization, Data curation, Formal analysis, Methodology, Project administration, Resources, Supervision, Visualization, Writing – review & editing. HW: Conceptualization, Data curation, Formal analysis, Funding acquisition, Investigation, Methodology, Project administration, Resources, Software, Supervision, Validation, Visualization, Writing – original draft, Writing – review & editing. JN: Conceptualization, Data curation, Formal analysis, Funding acquisition, Investigation, Methodology, Project administration, Resources, Supervision, Validation, Writing – review & editing.

## Glossary

ACI: Acute cerebral infarction
ELISA: Enzyme-linked immunosorbent assay
NIHSS: National Institute of Health Stroke scale
mRS: Modified Rankin Scale
TOAST: Trial of org10172 in acute stroke treatment
PAPP-A: Pregnancy-associated Plasma Protein-A
ProMBP: Proform of eosinophil major basic protein
MMP: Matrix Metalloproteinases
IGFBPs: Insulin-like growth factor binding protein
IGF: Insulin-like growth factor
APTT: Activated partial thromboplastin time
TT: Thrombin time
FIB: Fipinogen
PT: Prothrombin time
DD: D-Dimer
AST: Glutamic oxalacetic transaminase
ALT: Glutamic-pyruvic transaminase
LDL: Low-density lipoprotein
